# A case of acute non-typeable Hemophilus influenza infective endocarditis in a patient with hypocomplementemia

**DOI:** 10.1016/j.idcr.2023.e01756

**Published:** 2023-03-31

**Authors:** Parth Patel, Delvis Fogwe, Tarang Patel, Sachin Patil, Andres Bran-Acevedo, Yuji Oba, William Roland

**Affiliations:** a University of Missouri School of Medicine, Kansas City, USA; b Department of Internal Medicine, Mayo Clinic, Rochester, USA; c Division of Pulmonary and Critical Care Medicine, University of Missouri, Columbia, USA; d Department of Medicine, Division of Pulmonary and Critical Care Medicine, University of Missouri, Columbia, USA; e Department of Medicine, Division of Infectious Diseases, University of Missouri, Columbia, USA

**Keywords:** Infective endocarditis, Hemophilus influenzae, Hypocomplementemia, Invasive

## Abstract

Hemophilus influenzae is a gram-negative bacteria responsible for significant cases of invasive infections, especially in the pediatric population and in immunosuppressed adult patients. Before vaccination, most cases were frequently caused by capsulated or typeable variants. Due to the absence of effective vaccination against the nontypeable variant, it is now responsible for most invasive infections. Predisposing risk factors in adults include asplenia, hypocomplementemia, cancer, human immunodeficiency virus infection, and chronic cardiopulmonary disease. Immunity to the nontypeable variants causing disease is perplexing and not yet wholly described as they are genetically diverse. Infective endocarditis (IE) is a cardiac infection with devastating consequences if not detected earlier and treated appropriately. Gram-positive bacteria are the primary cause of IE overall, followed by gram-negative bacteria. Hemophilus species belong to the HACEK group of gram-negative bacteria responsible for causing IE in the pediatric population more than in adults. Hemophilus species, especially the nontypeable variant, is a rare cause of IE in adults. Here we present a case of IE due to Nontypeable Hemophilus influenzae in a 49-year-old caucasian male with hypocomplementemia.

## Introduction

*Haemophilus influenzae* (*H*. *influenzae*) is a gram-negative coccobacillus-shaped bacterium. The organism primarily inhabits the upper respiratory tract but can colonize the lower respiratory tract. *H. influenzae* is an exclusive human pathogen and may be either encapsulated (Typeable with six variable polysaccharide capsules) or unencapsulated (Nontypeable) [1], [2]. *H*. *influenzae* infections occur in children or immunocompromised individuals and most often result in otitis media, bacteremia, pneumonia, bacteremia, or meningitis [3], [4]. A rare but possible complication among Nontypeable *Haemophilus influenzae* (NTHI) infections is infective endocarditis (IE). The 'HACEK’ organisms, namely *Haemophilus*, *Actinobacillus*, *Cardiobacterium*, *Eikenella*, and *Kingella*, are commonly associated with IE, but of the subset, only around 5 % of cases are attributable to *Hemophilus* species [4]. Among this group, the vast majority are due to *H. parainfluenzae*, *H. aphrophilus*, and *H. paraphrophilus* infections [5]. NTHI subspecies IE comprises a minuscule number of rare *Hemophilus influenza* IE cases in adults. PubMed literature review revealed a single case of NTHI IE in a renal transplant patient [5]. Here, a case of IE secondary to an NTHI infection in a 49-year-old male with hypocomplementemia is described.

## Case presentation

A 49-year-old male with a medical history significant for previous intravenous (IV) methamphetamine use, active tobacco use, and *Neisseria meningitidis* bacteremia and meningitis (four months prior) was transferred to our facility for concerns of acute hemoptysis with pulmonary infiltrates. Before admission, the patient was seen at an outside hospital (OSH) and treated for an acute septic shock with chief complaints of nausea, vomiting, and diarrhea. Vitals revealed hypotension of 85/40 mmHg, bradycardia of 58 beats/min, a temperature of 97.5 F, and a respiratory rate of 18 breaths/minute. OSH labs demonstrated leukocytosis, thrombocytopenia, elevated creatinine, and hypoalbuminemia (Table 1). Computed tomography (CT) of the abdomen and pelvis demonstrated gastroenteritis and right-sided colitis. Initial blood cultures grew gram-negative coccobacilli, and the patient was started on IV vancomycin and piperacillin/tazobactam. He did not respond to fluid resuscitation and was started on vasopressors. The stool workup was positive for *C. difficile* colitis, and he was switched to oral vancomycin with improvement. The hospital course revealed septic shock resolution but was complicated by hemoptysis and a follow-up chest x-ray displaying pulmonary infiltrates. He was then transferred to our facility's medical intensive care unit for further evaluation. Upon arrival, he denied chest pain, palpitations, abdominal pain, fever, or chills. He affirmed productive cough (brown-colored phlegm) with dyspnea.

**Table 1:** Outside hospital laboratory results.

WBC = White blood cell count; CRP = C-Reactive Protein; ESR = Erythrocyte Sedimentation Rate; LDH = Lactate Dehydrogenase; HIV = Human Immunodeficiency Virus; MRSA = Methicillin Resistant *Staphyloccus aureus; C. difficile = Clostridium difficile.*

**Fig. 1:**
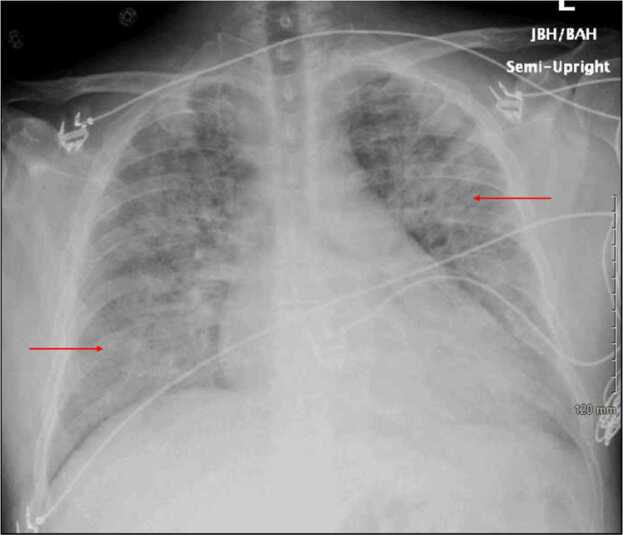
Chest X-ray portable revealed bilateral widespread patchy areas of airspace Opacification (Red arrows.

**Fig. 2:**
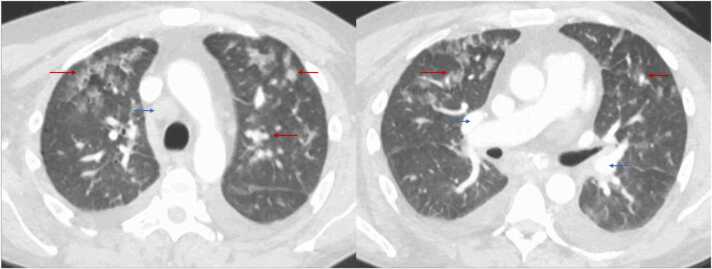
Computed tomography of the Chest with contrast revealed multifocal innumerable upper lobe predominant ground glass and nodular opacity (Red arrows) in bronchovascular distribution with mild pulmonary edema and reactive enlarged mediastinal and hilar lymph nodes (Blue arrows).

Clinical examination revealed him to be awake, alert, and well-oriented to time, place, and person with no focal neurological deficits. He had poor overall hygiene, extensive dental caries, regular heart rate and rhythm, and a grade 2/6 holosystolic murmur best heard over the left fifth intercostal space at the midclavicular line. Breath sounds were reduced bilaterally on the anterior chest fields, with rales appreciable in the upper and lower lung fields on auscultation. The abdomen was diffusely tender but not distended, and the extremities revealed no abnormalities. Labs revealed leukocytosis, thrombocytopenia, elevated creatinine, elevated inflammatory markers, elevated NT-proBNP, hypocomplementemia, nonreactive syphilis, human immunodeficiency virus, and acute viral hepatitis serology (Table 2). Although Antinuclear Antibody (ANA) was elevated, the lupus serology was negative without vasculitis or other autoimmune manifestations; his hemoptysis and creatinine elevation resolved spontaneously without steroids, making Anti-neutrophil cytoplasmic antibody (ANCA)-associated disease less likely. Chest x-ray demonstrated extensive patchy bilateral areas of airspace opacification (Fig. 1). CT scan of the chest with IV contrast displayed multifocal innumerable upper lobe predominant ground glass and nodular opacity in bronchovascular distribution and enlarged mediastinal and hilar lymph nodes (Fig. 2). OSH-positive blood culture samples were sent to the state microbiology laboratory and were confirmed positive for beta-lactamase-negative NTHI (Table 2).

**Table 2:** Laboratory results at our hospital.

WBC = White blood cell count; CRP = C-Reactive Protein; NT-proBNP = N-terminal (NT)-pro hormone B-type natriuretic peptide; ANA = Antinuclear Antibody; Ig = Immunoglobulin; HIV = Human Immunodeficiency Virus; OSH = Outside hospital.

**Fig. 3:**
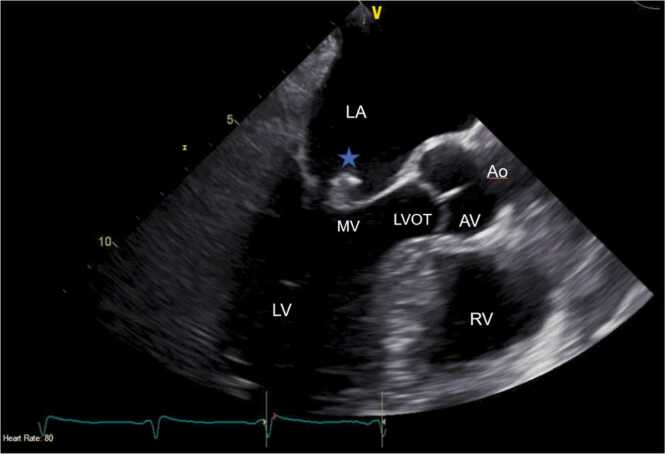
Transesophageal (TEE) ME LAX view revealing a large (1.73 × 0.55 cm), mobile vegetation (Blue star) on the atrial aspect of the anterior leaflet of mitral valve. LA = Left Atrium, LV = Left Ventricle, MV = Mitral Valve, RV = Right Ventricle, AV = Aortic Valve, LVOT = Left Ventricle Outflow Tract, Ao = Aorta.

**Fig. 4:**
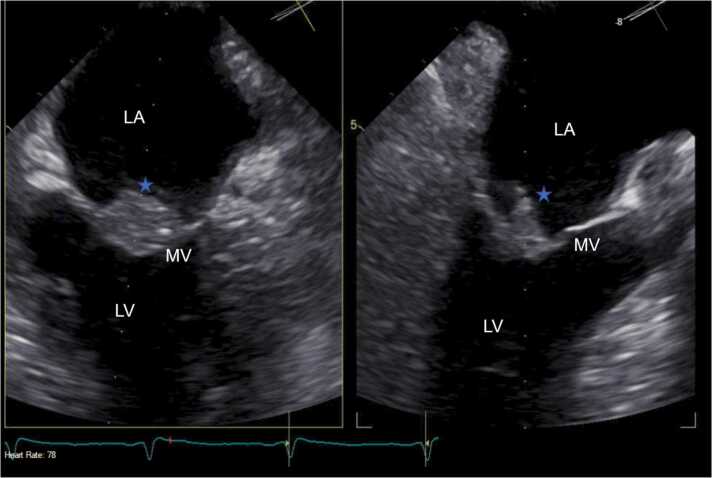
Transesophageal (TEE) Mitral commissural view revealing a large (1.73 × 0.55 cm), mobile vegetation (Blue star) on the atrial aspect of the anterior leaflet of mitral valve. LA = Left Atrium, LV = Left Ventricle, MV = Mitral Valve.

The infectious disease (ID) team was consulted for NTHI bacteremia. Antimicrobials were de-escalated to piperacillin-tazobactam for NTHI bacteremia and oral vancomycin for *C. difficile* colitis. As the chest x-ray revealed widespread pulmonary airspace opacities, a trans-thoracic echocardiogram (TTE) was ordered due to an IE concern. TTE demonstrated a normal ejection fraction, no wall motion abnormalities, and a 1.5 cm vegetation over the anterior mitral valve (MV) leaflet. A trans-esophageal echocardiogram (TEE) confirmed large mobile vegetation (1.73 × 0.55 cm) visualized on the atrial aspect of the anterior leaflet of the MV, which protruded to the left ventricle during diastole with severe mitral regurgitation (Fig. 3 & 4). After TEE confirmed IE, a panoramic x-ray of the oral cavity revealed multiple fractured maxillary teeth with extensive dental caries addressed by surgical extraction and alveoloplasty in all four quadrants. Based on ID team recommendations, IV ceftriaxone 2 gm every 24 h was started, and piperacillin-tazobactam was stopped. Cardiothoracic surgery (CTS) was consulted, and the patient underwent a bioprosthetic mitral valve replacement (MVR). Mitral valve histopathology revealed acute inflammation and necrosis consistent with IE. The patient completed six weeks of ceftriaxone inpatient after the MVR and was free of symptoms with a repeat chest x-ray revealing no acute cardiopulmonary abnormalities at a two-week CTS clinic follow-up. He was referred to a tertiary center for additional evaluation of his hypocomplementemia.

## Discussion

The average annual incidence from 2009 to 2015 of invasive *H. influenzae* infections has increased by 16 % from 1.47 to 1.70 cases per 100,000 population due to an increase in NTHI and capsular serotype a infections [6]. A significant epidemiological change post-Hib vaccine introduction has resulted in an increased incidence of NTHI and non b capsular serotype infections. Of all invasive *H. influenzae* infections, NTHI comprised 71.6 % of cases with a greater case fatality ratio (16.1 %) and the highest incidence (1.22). The incidence of invasive *H. influenzae* infections is highest in children aged < 1 year, followed by adults aged ≥ 65 with a median age of 64. In adults aged ≥ 65 years, the risk increases with advancing age due to NTHI disease. Common clinical presentations of invasive *H. influenzae* infections include bacteremia pneumonia (median age of 70 years), bacteremia (median age of 53 years), and meningitis (median age of 31 years) [6]. Clinical comorbidities in these patients include coronary artery disease, congestive heart failure, atrial fibrillation, diabetes, hypertension, dementia, chronic obstructive pulmonary disease, and smoking history [3], [6]. Recently, the incidence of HACEK bacteremia has markedly increased, with *Haemophilus* species accounting for a sizable portion (29 %) of all bacteremia. However, most of these result from *H. parainfluenzae* and *H. parahaemolyticus* rather than *H. influenzae* [7]. Thus, a notable difference in demographics and infectivity is present between *H. influenzae* and its other genus members.

IE is an exceedingly rare complication of an NTHI infection. In a compilation of 42 case studies on *Hemophilus*-derived IE, *H. influenzae* was found to be the causative agent in only three patients [8]. Additionally, in another report of 14 patients suffering from IE, only one was a result of *H. influenzae* [9]. Cases have been documented in which *H. influenzae* IE results in a fatal outcome; however, most of these cases result from typeable *H. influenzae* infections [10]. Nonetheless, the change in epidemiology and virulence factors have increased the incidence of NTHI infections. Before the *H. influenzae* serotype b (Hib) vaccine development, the Hib variant accounted for nearly 95 % of all invasive diseases and was the frequent cause of childhood meningitis and invasive *H. influenzae* disease [11], [12]. After successive Hib vaccine deployment, the incidence of invasive *H. influenzae* among young children dramatically decreased by > 99 %, creating a newfound niche for the non-b encapsulated and nontypeable variants [13], [14]. As a result, a noticeable epidemiological shift has occurred, and NTHI is now the leading cause of invasive disease in all age groups [13]. Due to the absence of an effective vaccine, infections by the NTHI have become increasingly invasive [13]. The NTHI development of more virulent attributes by NTHI has only compounded this phenomenon.

NTHI exhibit greater genotypic and phenotypic diversity and are genetically diverse than the encapsulated strains [15]. To avoid host detection, NTHI employs a host of immune evasion tactics. NTHI infections recur as the immune response (antibody) to NTHI strains is determined by their surface antigens. This specific immune response results in an inability of the host to prevent recurrent infections by different NTHI strains. Also, some colonized strains undergo antigen point mutation changes escaping the host immune machinery and causing an infection recurrence [4]. NTHI lacks a capsule, thus preventing host immune factor deposition on its surface [4]. Furthermore, NTHI can bind plasma proteins in the lung and manipulate the host through surface lipooligosaccharide interactions [16]. These attributes drastically decrease the effectiveness and efficiency of host complement-mediated killing [17]. NTHI has been demonstrated to produce biofilms in childhood otitis media infections that increase resistance to clearance [18] and enable subsequent relapse [16].

As observed in our patient, whatever minimal complement reaction occurred was further suppressed by the pathogen’s ability to resist complement-dependent killing, thereby increasing its virulence. Furthermore, most IE cases occur in immunocompromised individuals with underlying comorbidities. Among immunocompromised patients, hematogenous spread secondary to bacteremia from a different infection is the most common source of seeding heart valves [19]. This is the possible mechanism in our patient, as he was immunocompromised due to hypocomplementemia and presented with bronchoalveolar pneumonia due to NTHI. NTHI pulmonary infection was the source of his bacteremia and IE. Of note, our patient was relatively young compared to the average age of *H. influenzae* infections. Of the few IE cases secondary to the NTHI variant, the presentation is analogous to other causative microorganisms, including *S. epidermidis*, *H. parainfluenza*, and *H. aphrophilus* [20]. Prompt treatment of IE carries a good prognosis. Current guidelines recommend HACEK IE treatment to exclude penicillin and ampicillin and use ceftriaxone for four to six weeks. A fluoroquinolone is recommended in individuals with adverse reactions to ceftriaxone; ampicillin-sulbactam may also be used [4]. Due to the extensive mitral valve damage, our patient was treated with mitral valve replacement and IV ceftriaxone for six weeks.

## Conclusion

Clinicians should be aware of the rare but possible complication of IE in an NTHI sinopulmonary infection in patients. It is prudent to check adults with NTHI invasive infections for immune defects or other immunosuppressive causes. Appropriate history-taking and risk-factor evaluation for IE in patients with *H. influenzae* positive blood cultures are essential in obtaining an early diagnosis and avoiding further complications. As NTHI invasive infections are undergoing an epidemiological change, close surveillance is essential to monitor these trends for effective therapeutic management to avoid high-case fatality and mortality. The effectiveness of chemoprophylaxis in NTHI invasive infections is unknown; However, this should not prevent current efforts to develop a vaccine against the NTHI strains.

## CRediT authorship contribution statement

Parth Patel – Principal author. Delvis Fogwe – Co-author. Tarang Patel – Co-author. Sachin Patil – Faculty advisor and contributor. Andres Bran-Acevedo – Faculty advisor and contributor. Yuji Oba – Faculty advisor and contributor. William Roland – Faculty advisor and contributor.

Parth Patel, Delvis Fogwe, Tarang Patel, and Sachin Patil worked on drafting, editing, and reviewing the manuscript that was prepared for submission and performed a literature review for this project. Delvis Fogwe, Sachin Patil, and Andres Bran-Acevedo were involved in the patient's care being discussed. Sachin Patil, Andres Bran-Acevedo, Yuji Oba, and William Roland, as faculty advisors, contributed by assisting in reviewing and editing this case report and providing project supervision and administration. We confirm that all named authors have read and approved the manuscript. We further confirm that all have authorized the order of authors listed in the manuscript.

## Funding

There are no sources of funding to report for this case report.

## Conflicts of interest

The authors declare that they have no conflicts of interest.

## Data Availability

Not applicable
